# A spatial-temporal analysis at the early stages of the COVID-19 pandemic and its determinants: The case of Recife neighborhoods, Brazil

**DOI:** 10.1371/journal.pone.0268538

**Published:** 2022-05-17

**Authors:** Arthur Pimentel Gomes de Souza, Caroline Maria de Miranda Mota, Amanda Gadelha Ferreira Rosa, Ciro José Jardim de Figueiredo, Ana Lúcia Bezerra Candeias

**Affiliations:** 1 Programa de Pós-graduação em Engenharia de Produção, Universidade Federal de Pernambuco, Recife, Pernambuco, Brazil; 2 Departamento de Engenharia de Produção, Universidade Federal de Pernambuco, Recife, Pernambuco, Brazil; 3 Departamento de Engenharias, Universidade Federal Rural do Semi-Árido, Angicos, Rio Grande do Norte, Brazil; 4 Departamento de Engenharia Cartográfica, Universidade Federal de Pernambuco, Recife, Pernambuco, Brazil; Universita degli Studi del Molise, ITALY

## Abstract

The outbreak of COVID-19 has led to there being a worldwide socio-economic crisis, with major impacts on developing countries. Understanding the dynamics of the disease and its driving factors, on a small spatial scale, might support strategies to control infections. This paper explores the impact of the COVID-19 on neighborhoods of Recife, Brazil, for which we examine a set of drivers that combines socio-economic factors and the presence of non-stop services. A three-stage methodology was conducted by conducting a statistical and spatial analysis, including clusters and regression models. COVID-19 data were investigated concerning ten dates between April and July 2020. Hotspots of the most affected regions and their determinant effects were highlighted. We have identified that clusters of confirmed cases were carried from a well-developed neighborhood to socially deprived areas, along with the emergence of hotspots of the case-fatality rate. The influence of age-groups, income, level of education, and the access to essential services on the spread of COVID-19 was also verified. The recognition of variables that influence the spatial spread of the disease becomes vital for pinpointing the most vulnerable areas. Consequently, specific prevention actions can be developed for these places, especially in heterogeneous cities.

## Introduction

The World Health Organization (WHO) declared the coronavirus disease 2019 (COVID-19) pandemic in March 2020 [1]. It is a vascular disease caused by the severe acute respiratory syndrome coronavirus 2 (SARS-CoV2) virus, which perturbs multiple organ systems and prompts clinical manifestations in the lungs, heart, and kidneys, in particular [2,3]. In the early stages of the pandemic, the spread of the disease was mainly mitigated by non-pharmaceutical interventions [4], which were then complemented with vaccination programs [5] as technology evolved. However, changes of intervention measures [6] along with the emergence of highly contagious variants of the virus [7] have affected the rates and the patterns of the spread of COVID-19.

Spatial-temporal analysis might be a valuable tool to capture the dynamic behavior of the pandemic. One of the most regularly applied approaches in this context is spatial analysis, in which cluster and hotspot analysis, interpolation, and space–time scan statistics have been the main techniques used [8]. For instance, in China, spatial clustering patterns, over time, of cumulative cases of COVID-19 at the city [9] and county-levels [10] showed a shift from hotspots of larger coverage to few specific places. In global terms, evidence suggests that there is a sort of spread of COVID-19 cases from developed countries to less affluent countries [11].

South America saw its geographic centroid for COVID-19 cases generally shifting from west to east in Brazilian areas, up to January 2021 [12]. In Brazil, the centroids of cases and deaths started in São Paulo state (Southeast region), then progressively moved north until early May 2020 [13]. The COVID-19 epidemic increased rapidly across northeastern Brazil up to May 2020, at which time it was the metropolitan areas (mostly in the coastal areas) that were reported as having clusters of cases, even though there was an already relevant spread towards the countryside [14]. Understanding the transmission dynamics within local communities is important in order to develop strategies to combat the spread of the disease.

Additionally, another valuable analysis is investigating risk factors and determinants of COVID-19. Spatial regression tools have been used as a means to analyze the influence of these factors on the number of COVID-19 cases, incidence rate, the mortality rate and the case-fatality rate across space [15–17]. Some demographic indicators have been identified as crucial factors for the spreading of COVID-19, including the positive associations of cases of the disease with the elderly population [16,18,19], the percentage of people between 15 and 64 years-old [20], and the density of the population [16,19,21]. Different socio-economic factors are also found to positively impact COVID-19 incidence rates, such as income per capita [15], household income [16,22], life expectancy at birth [15], and the area deprivation index [18], whereas others affect them inversely including access to education [23]. Specifically, racial/ethnic minority relationships varied from positive with COVID-19 cases in regions of the USA [16,18] to negative with COVID-19 deaths in England [17]. In terms of health conditions, COVID-19 cases have positive associations with diabetes rates [16,19], while there are negative associations with smokers on the global scale [20] and with morbidity in South Korea [23]. Healthcare access [23] and available hospital beds [19] are also negatively related to the spread of COVID-19 cases. Finally, the built environment can be positively associated with COVID-19 cases with regard to the average height of buildings [21], and the density of commercial facilities and of roads [24], likewise negatively with the average street length [21].

Nonetheless, these relationships seem to vary according to the spatial scale, the study area and the phase of the outbreak [8]. On a global scale, population density, older populations, and household size are crucial predictors in early weeks of the reported spread of COVID-19, whereas the impacts of interpersonal contact and the globalization of trade are greater over time [11]. The mobility of the population also has a valuable role in this scenario, on varying spatial scales, since highly connected places [25] and areas with great service from bus stops and subway stations [26] showed a significant effect on the spread of the disease. Finally, an increase of mobility in the state of New York—due to the relaxation of restrictive measures—moved the COVID-19 clusters from counties of high population density to low ones [27]. Knowledge about the determinants and the community spread of the disease may support the development of specific sanitary strategies for the pandemic scenario. Strategies need to be created, evaluated, controlled and sustained by information on local characteristics.

In this regard, Brazil has been one of the most severely affected countries throughout the pandemic [12]. Brazil is a very large and complex country which is unequal in terms of public health and socio-economic matters, so there is not a unique explanation for the spatial spread of the virus across states [13]. Mortality risks tend to be higher for older men in Brazilian municipalities, whereas young adults and women are prone to higher risks of infection [28]. Also at the municipality-level in Brazil, a spatial regression model identified 13 indicators positively associated with the COVID-19 incidence rate, including the activity rate of people aged 10 to 14 years; the percentage of people aged six to 14 years who do not attend school; and the percentage of employed persons aged 18 or over who have completed elementary school [15]. In fact, the COVID-19 pandemic has been most severe in the poorest and most unequal regions of Brazil, such as the states of the Northeast region [6]. A recent study found that these states have suffered from a large number of infections and also a high mortality risk due to the deprived socioeconomic status of the poor and unsatisfactory health care conditions [28].

Considering the singular characteristic of the Northeastern states of Brazil, this study analyses the spatial patterns and driving factors of the spread of COVID-19 in the city of Recife, capital of Pernambuco state. So far, we found that previous studies about these topics in Recife were restricted to the association of the number of COVID-19 cases with the number of users of public transportation systems [29] and socio-economic factors [30]. Therefore, in this study, we adopted a three-stage methodology, in which we have investigated the spatial-temporal trend of the disease within the city and the impact on COVID-19 rates of local facilities that continued to function even during lockdown periods. Subsequently, we investigated the joint effect caused by socio-economic factors and the continuation of essential services on the spatial spread of COVID-19 confirmed cases and on the case-fatality rate on a neighborhood-level scale.

## Data and methods

### Design

**Fig 1:**
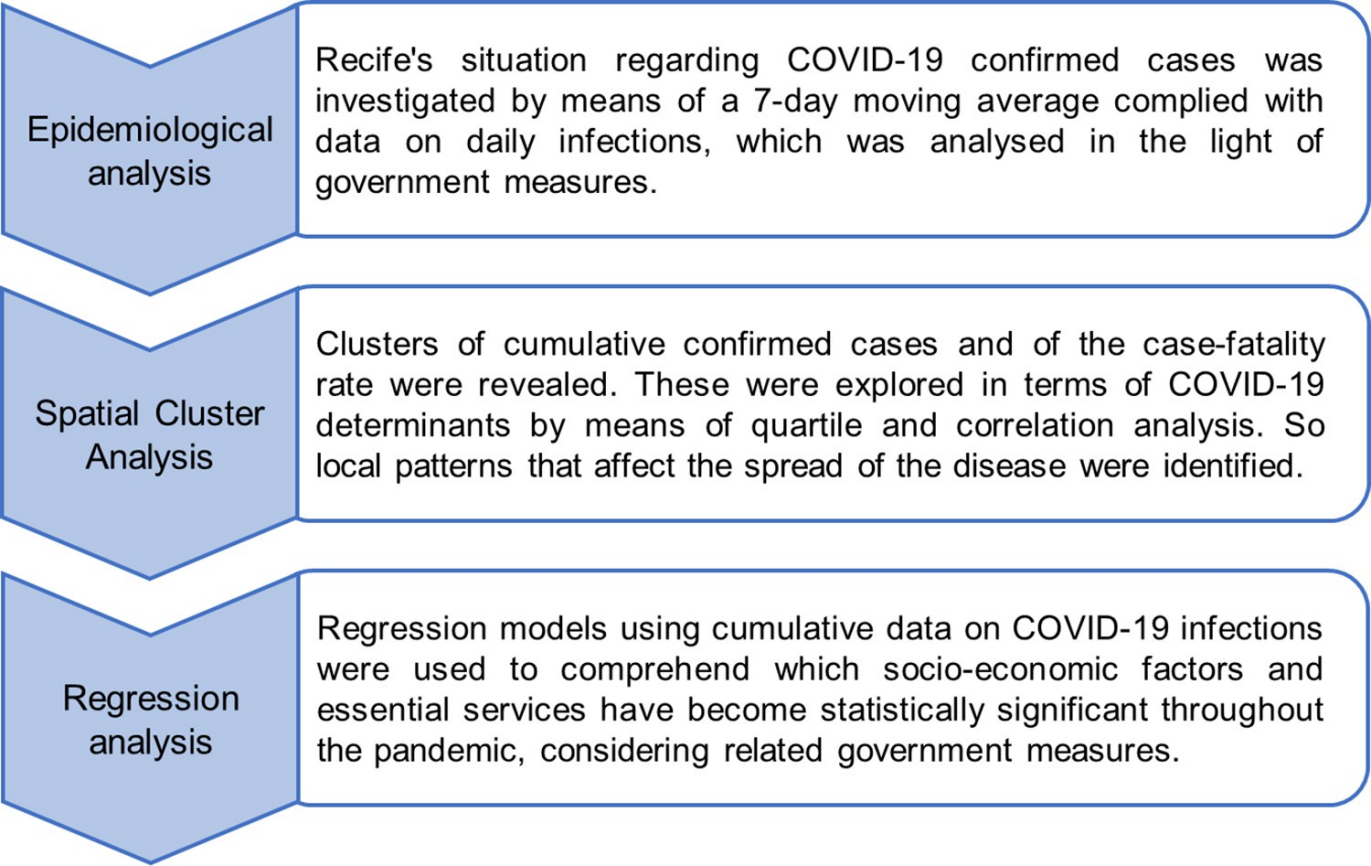
Ordered steps of the present study.

This study develops a spatial evaluation to comprehend the behavior of the outbreak of coronavirus across the neighborhoods of Recife, Brazil. Statistical approaches (correlation, quartile and regression analysis) were combined with GIS-based methods (hotspots and spatial regression analysis) for the purpose of exploring local characteristics that may make places more susceptible to the spatial spread of COVID-19. A brief summary of the methodology is set out in Fig 1.

**Fig 2:**
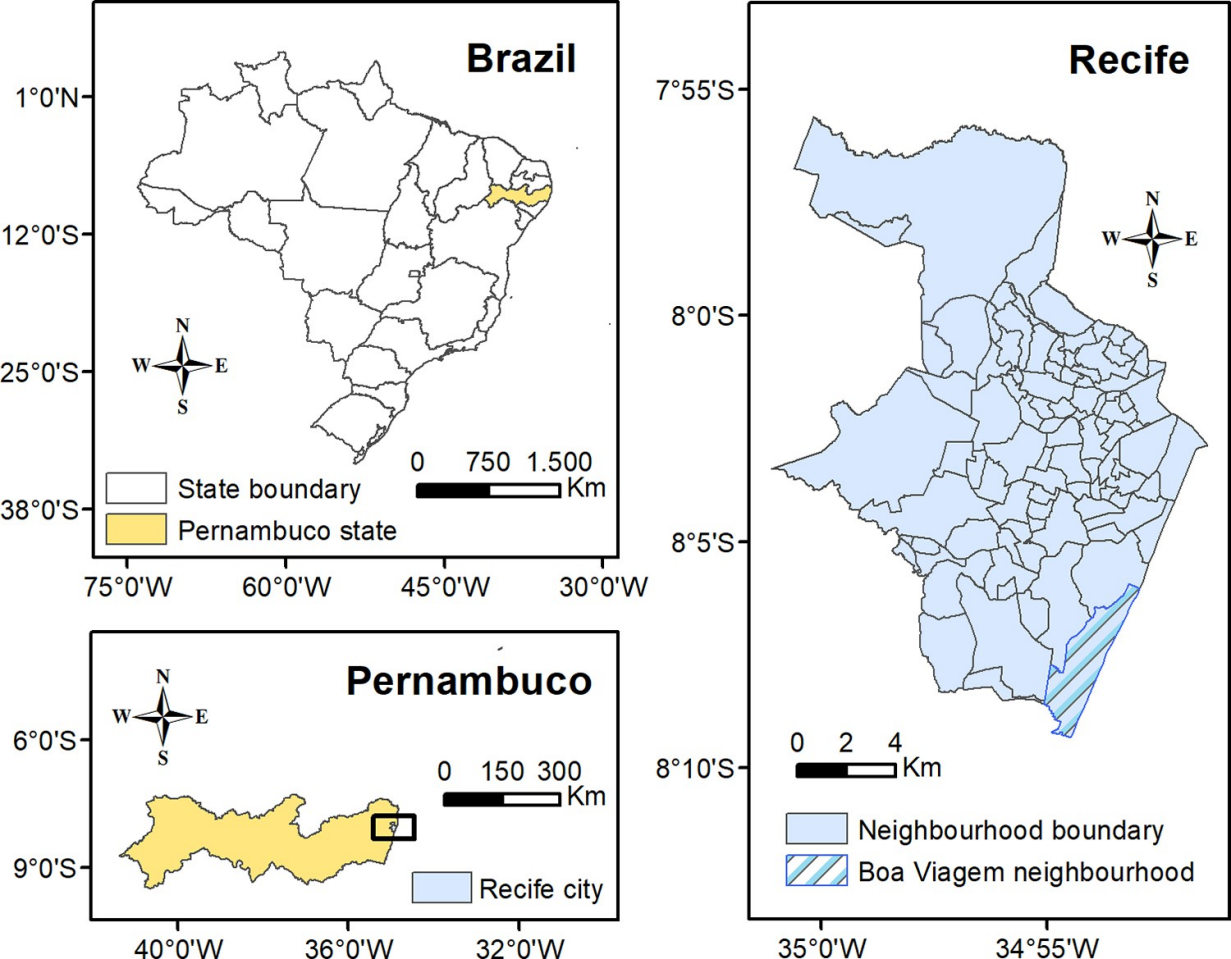
Location of the study area. Sources: Brazilian Institute of Geography and Statistics (IBGE) 2021, and software ArcGIS 10.4.1.

The city of Recife is the capital of the third most populated state in the Northeast region of Brazil (Pernambuco). Recife covers an area of 218 km^2^ and the estimated population is 1.55 million [31]. The city is located on the coast, in the Southern Hemisphere, and below the Equator. Recife has a tropical and humid climate, with an annual average temperature of about 26°C and a small variation of 5° C, approximately. It has the highest GDP per capita and is the second-most densely populated city in NE Brazil [31]. Moreover, the city had the highest Municipal Human Development Index (MHDI) among Northeast state capitals in 2010 (0.772), but it had just the 13th best performance out of 27 state capitals and was the 210th best city index nationally [32]. On the other hand, in 2019, Recife had the highest Gini index (0.612) among Brazilian state capitals, which reveals its heterogeneity and the extreme inequality of the distribution of income [33]. For instance, some neighborhoods are considered wealthy, but they also contain sizeable favelas. In some cases, a neighborhood is surrounded by others with almost the opposite socio-economic conditions even although they are in the same zone of the city [34]. There are two zones in Recife which have a very high HDI: one in the North and the other in the South. The latter mainly covers the Boa Viagem neighborhood. On the other hand, two-thirds of the districts (namely 42 out of 62) are classified as having either a low or average HDI [34]. Fig 2 shows the location of Recife and its neighborhoods.

### Data collection

The numbers of reported cases and data on deaths were obtained from the Planning and Management Secretariat of the state of Pernambuco, Brazil [35]. Daily records on Recife were provided at the city-level, but there was a lack of reliable data at finer scales. Georeferenced data were available only at the neighborhood-level, from 16 April to 3 July 2020, in cumulative form, and were published approximately once a week.

Socio-economic and demographic factors were selected to represent the main characteristics of the neighborhoods in terms of population structure and living conditions. In this regard, a set of 15 census indicators was extracted from the 2010 Brazilian Census [36], and is described in Table 1. We extended this set to characterize the impact of the presence of non-stop services (the so-called essential services) during the pandemic, since their locations have the potential for crowds to form and for premises to become overcrowded. So, six environmental facilities—that represent each of these services—were considered based on the classification proposed by the Planning and Management Secretariat of the state of Pernambuco, Brazil [37]. Georeferenced point data concerning these places were extracted from Google Maps and Google Earth platforms. These facilities are spatially described in Table 2 by means of Kernel density estimation [38]. However, they were analyzed throughout the paper in the form of counting in the polygons that delimit the neighborhoods of Recife.

**Table 1:** Descriptive statistics of census indicators.

Indicator	Max	Min	Mean	Standard deviation	Definition
Income (R$)	10000.00	510.00	2054.50	2131.17	Average household income per month
Population	122922	72	16358.55	18274.11	Total of residents per neighborhood
RPH	4.5	1.73	3.25	0.30	Residents per household
Literacy (%)	0.9789	0.7159	0.8627	0.0533	Literate residents over 6 years old
Piped water (%)	0.9954	0.1250	0.8367	0.1709	Households supplied with piped water
Sewage disposal (%)	1.0000	0.0484	0.5628	0.2930	Households supported by sewer network
Electricity (%)	1.0000	0.9226	0.9979	0.0080	Households supplied with electricity
Garbage collection (%)	1.0000	0.7346	0.9755	0.0438	Households served by garbage collection service
Owned home (%)	0.8687	0.4577	0.7268	0.0695	Homes owned by their residents
Rented home (%)	0.5141	0.1111	0.2231	0.0648	Homes rented by their residents
Age 0 to 9	12149	6	2147.05	2306.98	Residents separated by age group per neighborhood
Age 10 to 19	15129	0	2613.43	2791.56	
Age 20 to 39	41556	30	5649.29	6325.24	
Age 40 to 59	33813	15	4015.44	4653.87	
Age over 60	20275	9	1933.23	2479.59	

The data regarding COVID-19 rates and their explanatory factors (including the facilities) were joined to the administrative boundary shapefile of Recife neighborhoods obtained from the Brazilian Institute of Geography and Statistics (IBGE) (https://downloads.ibge.gov.br/) using ArcGIS 10.4.1. This software was also used to produce the maps.

**Table 2:** Description of essential services and their spatial density.

Essential service	Definition	Spatial density of the essential services
Bakeries	Shops where baked goods are made and sold	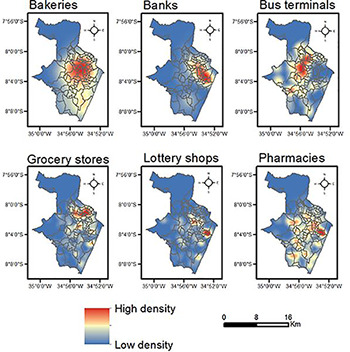
Banks	Financial institutions licensed to provide services such as receiving deposits and making loans	
Bus terminals	Places formed by waiting areas, stands for buses and ticket offices where buses start and end their routes	
Grocery stores	A range from small shops to large supermarkets which sell food and general items for domestic use	
Lottery shops	Official banking correspondents that provide financial services, receive payment of utility bills, pay social protection benefits, and sell lottery products	
Pharmacies	Stores where medicinal drugs are sold or given out	

Sources: Brazilian Institute of Geography and Statistics (IBGE) 2021, and software ArcGIS 10.4.1.

### Data analysis

An initial investigation presented Recife’s epidemiological situation regarding confirmed cases over time starting with when the first cases were confirmed in March 2020. A 7-day moving average was applied to the data on daily infections at the city-level, thereby seeking to reduce sudden variability due to reporting biases such as the lack of testing and the delay in recording cases and deaths [39]. The impact of restricting and relaxation measures imposed by the State Government of Pernambuco and the City Council of Recife on the variability of the rate of new infections was also discussed. These government actions include the following: closing of non-essential commercial activities; mandatory use of masks; strict quarantine; reopening of building supply stores, beauty salons, suburban retailers, malls, and places of worship [37].

In order to comprehend disparities in the spread of COVID-19 on a small scale, neighborhoods were explored using a spatial cluster analysis as applied for other diseases [40,41]. Georeferenced data obtained from ten specific days between 16 April and 3 July 2020 at intervals of roughly one week were considered. Nevertheless, we were able to represent the ascending, peaking and descending behaviors of the curve of infections.

At first, for each of the ten dates, we checked if the cumulative confirmed COVID-19 cases and the case-fatality rate (which means the total number of deaths divided by the total number of cases) were spatially dependent across the study area. In this regard, the spatial statistic Global Moran’s I [42,43] was performed considering spatial weights obtained from inverse distance and contiguity of edges and borders among neighborhoods. Then, Local Moran’s I [44] was implemented for cumulative confirmed cases and the case-fatality rate—which means the total number of deaths divided by the total number of cases. This statistic assessed the spatial autocorrelation associated with each neighborhood of the study region in terms of a few surrounding spatial units. It was calculated for each area *i* = 1,…,*n* in:

$$I_{i}=\frac{\sum_{j=1}^{n}W_{ij}(y_{i}-\bar{y})(y_{j}-\bar{y})}{\frac{1}{n}\sum_{j=1}^{n}\left(y_{j}-\bar{y}\right)}$$ (1)

where *y* means the COVID-19 reported cases or case-fatality rates for the *i*th area or its *j*th neighboring areas, and *W*_*ij*_ means a weight that represents proximity for the pair of areas *i* and *j* (in this case, the inverse distance between them). As a result, the location of statistically significant spatial clusters and outliers could be identified, represented in maps and further explored. These clusters are called High-High (high values surrounded by high values; or hotspots) and Low-Low (low values surrounded by low values), whereas the outliers are the High-Low (high values surrounded by low values) and Low-High (low values surrounded by high values) entities [45].

We also investigate patterns of driving factors within spatial clusters and outliers to understand the spread of COVID-19 in the study area. Thus, we emphasized distinctive community characteristics associated with highlighted clusters, at different stages of the pandemic. The investigation of the determinants aims to test if the pattern of the spread of COVID-19 in Recife follows the global pattern [11]. Moreover, we explore the impact of the local demography, expecting that the population [16,19,21] and the economically active population [20] factors would be highly associated with the occurrence of the disease. Finally, as COVID-19 cases are positively associated with the density of commercial facilities [24], we expect an analogous behavior regarding the presence of essential services.

For this purpose, 15 socio-economic factors were submitted to quartile analysis, and Spearman’s rank correlation tests were conducted in relation to case-fatality rates only. Additionally, the relevance of the dataset of 6 essential services was ratified due to its linear connection with case-fatality rates noticed in scatterplots, which justified the use of a Pearson’s product-moment correlation.

Subsequently, we have performed several regression analyses. Regression methods are widely applied in health-related studies [15–22,46,47]. The Ordinary Least Squares (OLS) regression technique [48] was used to reveal the strength of the relationships between the dependent and the most significant explanatory variables throughout different stages of the pandemic. A regression analysis was applied using as a dependent variable only the cumulative data on COVID-19 infections gathered from the latest date then available, July 3^rd^, 2020. To further our exploratory concern, initially, essential services and socio-economic factors were treated separately, thus assuring that relevant relationships among explanatory variables from each dataset would not be discarded. An OLS regression was run twice, one per dataset. Correlated variables were eliminated when they had a variance inflation factor (VIF) greater than 7.5 as previously verified in other COVID-19 health studies [49,50]. We used a stepwise method based on the Akaike Information Criterion (AIC) in order to reduce both sets of determinants to their non-redundant cores [51].

An extended investigation was undertaken by performing regression analysis for both datasets, socio-economic and non-stop services facilities, as a way of understanding their likely synergistic effect for predictions. The OLS method was adopted, but now considering the same ten dates from which georeferenced data on infections at the neighborhood-level were available. The reason is to understand what determinants became statistically significant over time as the pandemic evolved and government measures were in force. In this study, multi-source data can be used since we have complementary full datasets for the populations studied [52], on which a one-to-one linkage procedure was applied for complete observations, instead of sample-based datasets [53].

A k-fold cross-validation procedure [54] was applied to evaluate possible overfitting of the OLS regression models. The dataset of COVID-19 cases regarding the neighborhoods was split into randomly selected k = 5 folds of similar size. A fold of the data was held-out for final validation (testing), while the remaining k-1 formed the training set (learning). For these remaining subsets, k-1 iterations of training and validation were performed on them and the RMSE (root mean square error) was measured for each fold. The average RMSE of these iterations was compared to the same metric of the initially held-out fold. If the results are found to be close according to the context, i.e. a maximum difference of around 4 units, then there is no significant overfitting.

We are dealing with geospatial data, so Geographically Weighted Regression (GWR) [55] was used as a way of relaxing OLS assumptions that the observations and the error terms are independent and constant over the study region [45]. GWR takes into account the spatial autocorrelation and allows relationships among variables to vary over space and to be determined for each location [55]. In this method, a kernel function with a bandwidth parameter is used to calculate a local weights matrix in terms of the distance between each pair of spatial units [56]. Considering *i* = 1,…,*n* as each sample location, GWR is mathematically denoted by [56]:

$$y_{i}=\beta_{i0}+\sum_{k=1}^{p-1}\beta_{ik}x_{ik}+\varepsilon_{i}$$ (2)

where *y*_*i*_ is the dependent variable of COVID-19 confirmed cases at neighborhood *i*, *x*_*ik*_ is the value of the kth explanatory variable at location *i*, *β*_*i*0_ is the intercept, *β*_*ik*_ is the regression coefficient for the kth explanatory variable, *p* is the number of regression terms, and *ε*_*i*_ is the random error at location *i*. Cumulative count data has been applied in studies of COVID-19, especially at a small-scale level such as neighborhood or county-level [57] and grid [58]. The study of [59] predicted cumulative confirmed and cured cases of COVID-19 at a province-level, while [60] considered the number of deaths. Furthermore, the use of count data as a dependent variable instead of some sort of rates at the neighborhood-level is advocated by [61], and it is widely applied in the context of studies of crime [62].

OLS and GWR statistical performances were compared, thereby taking account of the reduced set of determinants found by using data acquired from July 3^rd^, 2020. Then we determined how GWR outputs explain the spread of COVID-19 in each neighborhood, and we also elucidated how every relevant factor impacts on the prominence of hotspots for new cases. According to the spatial context, an adaptive Gaussian kernel function was chosen with a view to adjusting the weighting for the density of infections. The kernel bandwidth parameter was estimated by means of a corrected Akaike Information Criterion (AICc) approach, based on which the optimal number of neighboring areas was found. Finally, in order to check if there is no spatial dependence on GWR residuals, Global Moran’s I [42,43] was applied to them. The weighting was once again based on the contiguity of edges and borders among neighborhoods.

Spatial analysis was conducted on ArcGIS 10.4.1 software, while the statistical tests and OLS regressions were performed on the R 3.6.1 platform.

## Results

**Fig 3:**
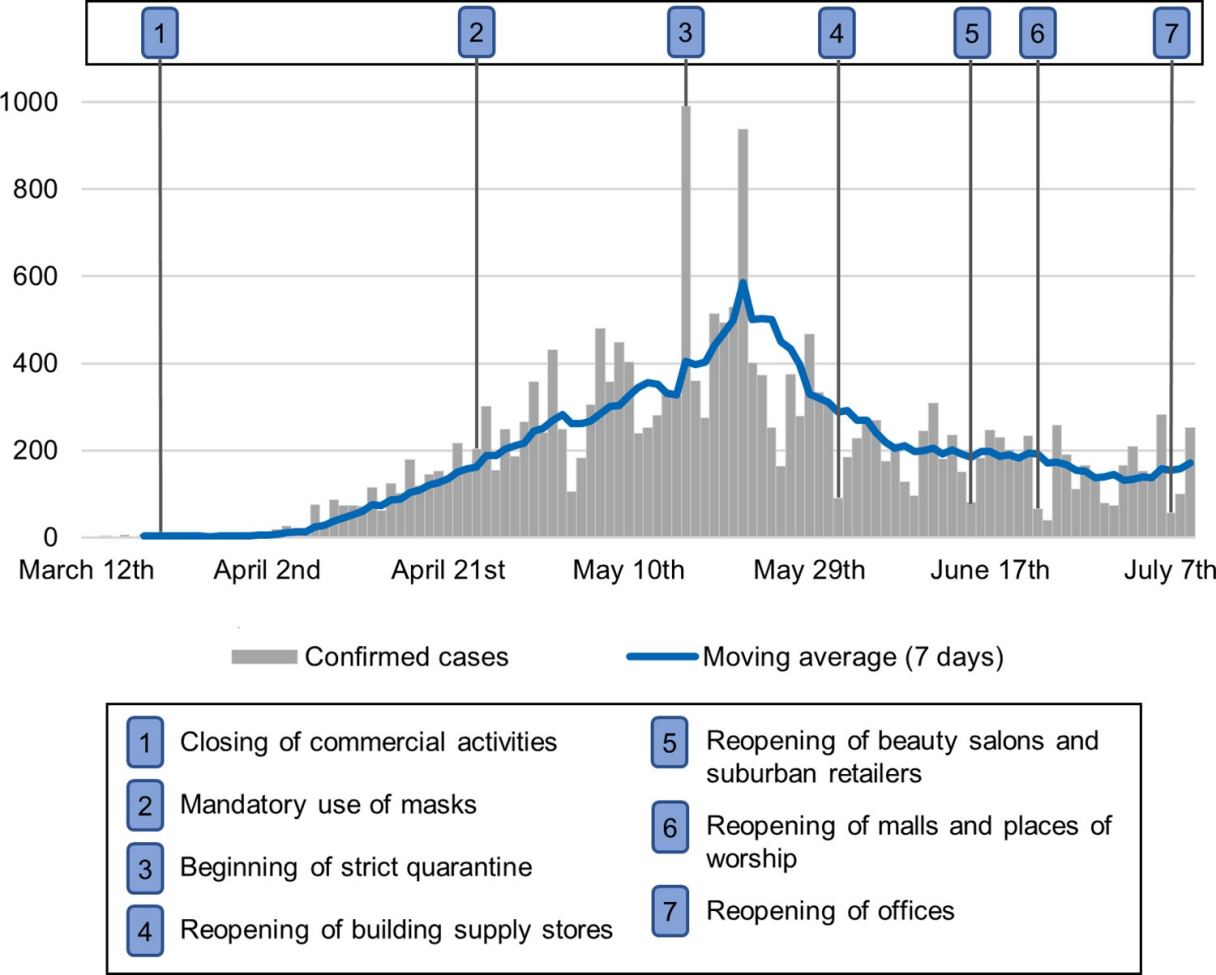
Evolution of daily COVID-19 cases in Recife from March 12^th^ to July 8^th^, 2020.

An evolution of cases over time in the city of Recife was plotted on Fig 3, including relevant actions taken by the State Government and the City Council. It was noted that these authorities took an early decision to close facilities when first cases were reported. Only a few types of facilities were allowed to open, including supermarkets, grocery stores, bakeries, and pharmacies [37]. As daily infections were increasing, the state governor issued an edict obliging the population to wear masks in public places. However, as fines were not imposed on shoppers but on the owners of the commercial facilities they entered, adherence to the measure became more dependent on the willingness of the general public to wear masks and intense supervision at the entrances to commercial premises.

These measures were not sufficient to flatten the increase in cases of infection, so in mid-May, a 15-day strict quarantine was imposed in five municipalities in Pernambuco, including Recife. People were only allowed to leave their homes to seek essential services, for which they had to show proof, and vehicles were only allowed on roads according to a vehicle rotation system based on the final number of the number plate [34]. Transmissions of the virus peaked in late May 2020 and the cases seemed to have been stabilized at a low level for that moment (July 8^th^, 2020), even though restrictions were then gradually relaxed and places in which people congregate such as shopping malls and commercial premises were reopened.

### Spatial clustering analysis of cumulative COVID-19 cases and case-fatality rate

A significant spatial dependence (p < 0.05) was found in Recife neighborhoods for the case-fatality rate on the majority of dates, according to Global Moran’s I, with an increasing tendency of significance as the pandemic evolved. On the other hand, the clustering of reported cases was significant for the first date analyzed and the last two dates. Consequently, spatial clusters for cases and the case-fatality rate tend to be formed across the study region.

**Fig 4:**
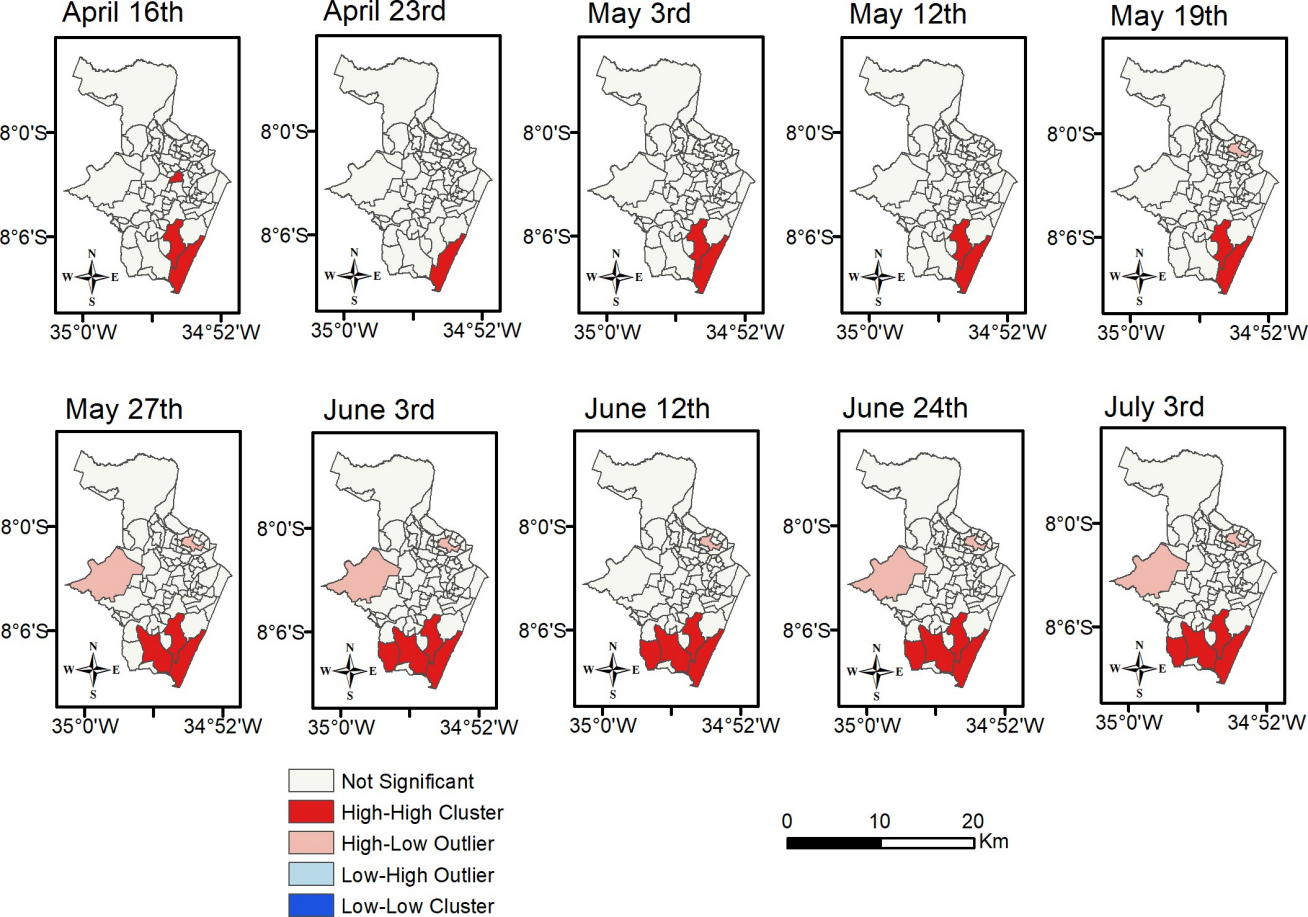
Spatial distribution of cumulative reported COVID-19 cases in Recife at the neighbourhood-level. Sources: Brazilian Institute of Geography and Statistics (IBGE) 2021, and software ArcGIS 10.4.1.

The locations of these clusters were found by means of Local Moran’s I. The results for the total number of reported cases per neighborhood are shown in Fig 4. We noted that cases of the disease at first were concentrated in the South Zone of the city, specifically in the neighborhood called Boa Viagem (Fig 1). Furthermore, it was the only significant spatial cluster on April 23^rd^, 2020. As cases increased, other High-High clusters and High-Low spatial outliers were found in the South, the North and the West Zones of the city.

Moreover, a quartile analysis was developed in the entire city using the 15 census indicators [36] that are listed and described in Table 1 (see Section 2 above). This analysis concerned the socio-economic and demographic factors that define the residents’ environment in terms of average income, population size, extent of home ownership, government support and by age group. Results revealed social disparities in the clusters shown in red in Fig 4 compared to Boa Viagem. While Boa Viagem is highly populated and yet well-developed in terms of its access to sanitation, garbage collection, and of levels of income and literacy, the other hotspots were found to have indices for these variables that were lower than at least 50% of all other neighborhoods in Recife.

**Fig 5:**
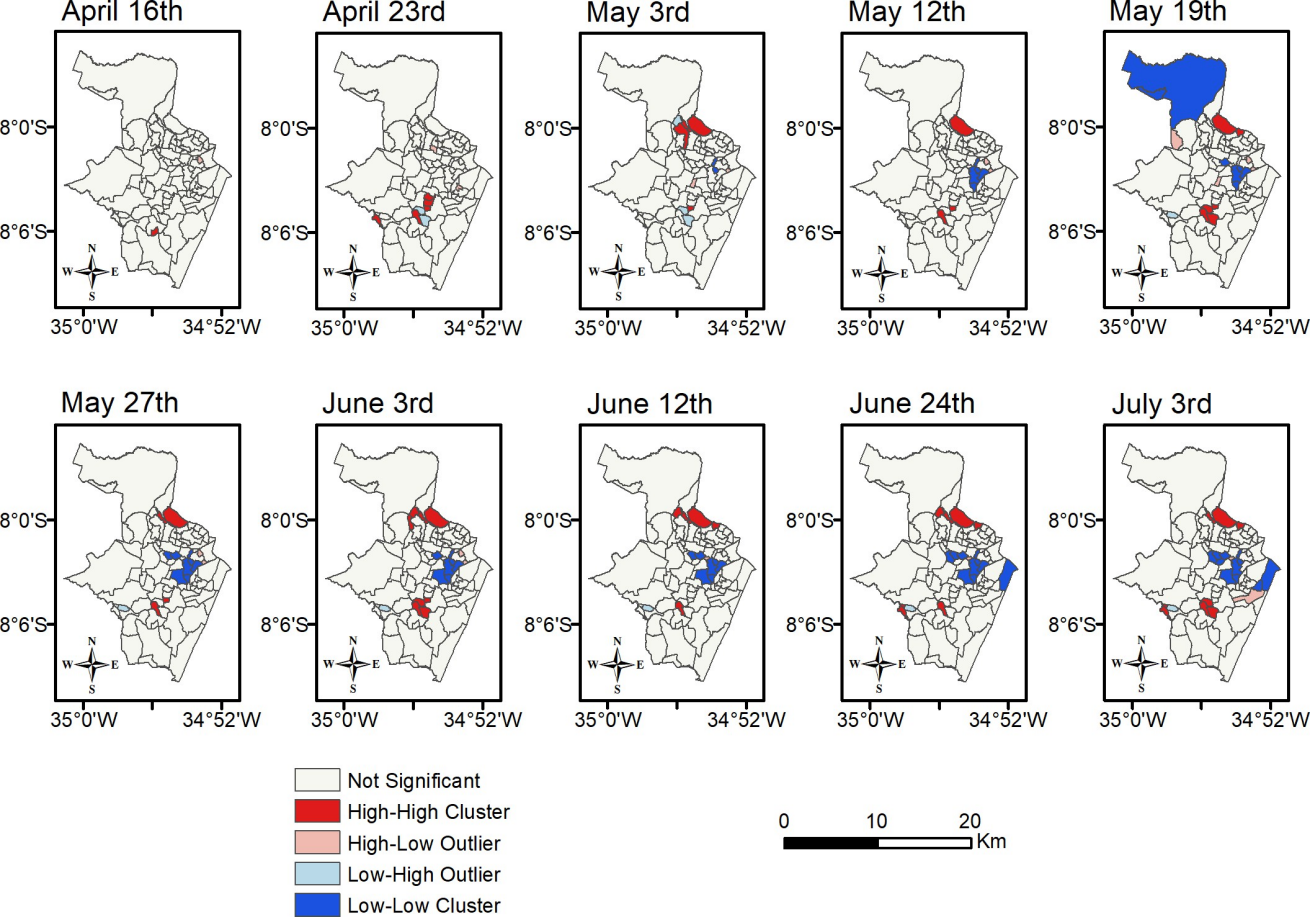
Spatial distribution of case-fatality in Recife as a result of cluster analysis.

Case-fatality rate (CFR) hotspots due to COVID-19 were also examined as plotted and shown in Fig 5. A different spatial pattern arises when these are compared to the clusters of cases. Over time, the CFR ones remained mostly stable so that High-High clusters usually formed in the North and Southwest zones, while Low-Low ones were seen in the North and West zones.

Even though Boa Viagem has been a significant cluster for reported cases since the first date analyzed, this has not occurred for the case-fatality rate. This neighborhood presents a high number of cases, but the CFR has been growing in a lower proportion. The first death in Boa Viagem was confirmed on April 10^th^, 2020 after 63 cases had been recorded, which represented 15% of the COVID-19 cases in Recife at that time. Boa Viagem led the number of deaths in the city from April 27^th^ until July 8^th^, 2020. However, this neighborhood did not become the hotspot of CFR in any of the transitional periods, i.e., Boa Viagem had the highest number of cases but it has had a low number of deaths per cases. On July 3^rd^, 2020 the neighborhood reached its maximum CFR, 18%, which, however, is considerably lower than the hotspots of CFR, for which the percentages were between 34% and 54%.

The results from quartile analysis show that hotspots of case-fatality rate usually present areas with similar environmental characteristics to those in hotspots of confirmed cases, disregarding Boa Viagem. For instance, most of these areas have a precarious public service provision and a low-income population. The opposite characteristics were verified when analyzing the Low-Low clusters. It has also been observed that neighborhoods in Low-Low clusters (of both case and case-fatality rate) have fewer residents per household than 75% of all other neighborhoods.

Spearman correlation tests were applied to the case-fatality rate and socio-economic factors using data from July 3^rd^, 2020. We detected that CFR is positively associated with residents per household, whereas it is negatively associated with household income, the literacy rate, access to the sewage system and to garbage collection, and the total of people over 60 years old.

Comparing previous results, we noted that some places tend to suffer from fewer deaths due to COVID-19 when residents in such places have a level of income and literacy that is well above average for Recife. These wealthier areas also have more access to public services and their number of residents per household is lower than elsewhere in Recife. In contrast to what was expected, the results also showed that the elderly population was higher in Low-Low clusters of case-fatality. In other words, although the elderly are more prone to catching severe forms of COVID-19 [63], the incidence of such cases was less in clusters with a high concentration of this population.

We also examined the presence of essential services (Table 2) in spatial clusters of case-fatality from July 3^rd^, 2020 by means of Pearson’s product-moment correlation. After exploring the Low-Low clusters for the case-fatality rate, the ‘number of banks’ was the only factor significantly associated with the CFR (negative). Six out of ten neighborhoods were placed in the fourth quartile of this determinant.

### Association between environmental factors and COVID-19 cases

Local determinants for COVID-19 were explored in greater depth by using spatial regression analysis. A set of 15 explanatory variables was compiled from census indicators (Table 1), whereas places that are typically conducive to attracting crowds of people and that operated even during strict quarantine (Table 2)—and so could have become centers of SARS-CoV2 infections—formed another set.

As an initial exploration, the determinants were processed separately in two regression models, according to their datasets of origin. We used the cumulative data of COVID-19 confirmed cases from July 3^rd^, 2020 as a response variable in each model based on the OLS method and reduced the number of determinants to non-redundant sets. Both regression models were significant (p < 0.001). With regard only to essential services, bakeries, grocery stores, banks and pharmacies remained in the final set of determinants with a high adjusted R^2^ of 0.8659. It was noted that ‘bakeries’ is associated with 77% of the variability in confirmed cases. Considering only the socio-economic factors, five determinants formed the significant final set (R^2^ of 0.9521): ‘people between 0 and 9 years old’ and ‘people older than 60 years old’ (extreme opposites among the age groups), ‘piped water’, ‘garbage collection’ and ‘residents per household’. The total of residents (factor ‘population’) is associated with nearly 90% of the variability in COVID-19 cases.

New assessments were made based on an aggregated set of 21 driving factors, regardless of the origins of this data, to understand how they can interact throughout space. OLS regression models were built for ten different days between April and July 2020, taking the cumulative reported cases of COVID-19 as the dependent variable. A smaller and significant set of explanatory variables was identified for each day by excluding correlated variables and using the stepwise method. Findings are summarized in Table 3 and, although determinants were analyzed together, the final sets were shown separately to clarify patterns. The column called GM, government measures, indicates which decisions were being imposed on each date by local authorities in their attempt to gradually reduce the number of people on the streets or to enable more people to be on the streets.

**Table 3:** The performance of OLS models over time using combined datasets of determinants.

Date	GM ^a^	Essential services	Socioeconomic factors	Adj. R^2^
April 16^th^	1	Bakeries, grocery stores, lottery shops, bus terminals	Owned home, income	0.8117
April 23^rd^	1; 2	Bakeries, grocery stores, banks	Age 0 to 9, owned home, sewage system	0.8159
May 3^rd^	1; 2	Bakeries, grocery stores	Age 0 to 9, income	0.8331
May 12^th^	1; 2	Bakeries, grocery stores, lottery shops, bus terminals	Age 0 to 9, owned home, income, literacy	0.8689
May 19^th^	1; 2; 3	Bakeries, grocery stores, lottery shops	Age 0 to 9, income	0.8958
May 27^th^	1; 2; 3	Bakeries, grocery stores, lottery shops	Age 0 to 9, income	0.9100
June 3^rd^	2; 4	Bakeries, grocery stores, lottery shops	Age 0 to 9, income	0.9162
June 12^th^	2; 4	Bakeries, grocery stores, lottery shops	Age 0 to 9, owned home, literacy	0.9215
June 24^th^	2; 4; 5; 6	Bakeries, grocery stores, lottery shops	Age 0 to 9, owned home, literacy	0.9255
July 3^rd^	2; 4; 5; 6	Bakeries, grocery stores, pharmacies	Age 0 to 9, income	0.9260

^a^Government measures in 2020: 1. Closing of non-essential commercial activities; 2. Mandatory use of masks; 3.Strict quarantine; 4. Reopening of building supply stores; 5. Reopening of beauty salons and suburban retailers; 6. Reopening of malls and places of worship.

Every designed model was found significant (p < 0.001). The adjusted determination coefficient R^2^ value reached a high level from the first date analyzed. As time progressed in the pandemic, the correlation of demographic and socioeconomic factors to cumulative cases increased, which prompted the same pattern for the adjusted R^2^. For instance, evaluating the contribution of each determinant in the final model (Table 3) separately in April 16^th^, ‘bakeries’ is associated with 76% of the variability in cases, followed by lottery shops (47%) and ‘grocery stores’ (39%). This same procedure was conducted in July 3^rd^, resulting in ‘children from 0 to 9 years old’ being associated with 80% of the variability in cases, followed by ‘bakeries’ (77%) and ‘pharmacies’ (73%). However, this increasing tendency for the adjusted R^2^ remained only until early June 2020, and was followed by a stabilisation pattern. It is likely this change was due to the scenario of the slow growth of cases in Recife as seen in Fig 3.

Even though the adjusted R^2^ metrics are high, it is not justified by overfitting. This hypothesis was discarded after each OLS model had been cross-validated based on partitions of the COVID-19 cases dataset and a comparison of RMSE (root mean square error) indexes. As ‘bakeries’ and/or the children’s age group are part of the models from the first date analyzed, they appear to be one of the main sources of the high adjusted R^2^. Other determinants are frequently repeated in the final sets, including ‘income’ and ‘grocery stores’, which reveals their importance for predicting cases. Furthermore, the variables ‘people older than 60 years old’ and ‘population’ (both highlighted in the initial analysis using separated databases) were disregarded after reducing the set of determinants to their non-redundant cores. This likely happened due to their strong collinearity with ‘bakeries’, namely a Pearson’s correlation of 0.85 (p < 0.001) for the total of residents and 0.93 (p < 0.001) for the elderly. Conclusively, all of the highlighted determinants in Table 3 (except for ‘bus terminals’) and the factors ‘population’ and ‘people aged over 60’ are positively associated with COVID-19 cases.

#### Spatial associations between environmental factors and COVID-19 cases

Data from the last day from which there is available georeferenced data at the scale studied, July 3^rd^, 2020, were kept for a subsequent evaluation using a spatial regression approach, GWR. So, the following set of relevant explanatory variables was considered: number of grocery stores, number of pharmacies, number of bakeries, the average income of residents and the total number of residents aged 0 to 9 years old. We used the cumulative confirmed cases as the dependent variable. According to the AICc approach, the optimal number of 54 neighboring areas per neighborhood was taken as the bandwidth parameter to compute the spatial weights. Finally, the GWR model resulted in a R^2^ of 0.960. A Global Moran’s I test applied to GWR residuals showed an index of -0.028 (p = 0.63), which provided evidence that the residuals are randomly distributed, and thus the model is adequate. Note that there is a slight improvement from the OLS results (global analysis) to those of the GWR (local analysis) since adjusted R^2^ increased from 0.926 to 0.944. Also, AIC statistics reduced from 935.99 to 916.19, indicating a significant improvement in the quality of the model [64].

**Fig 6:**
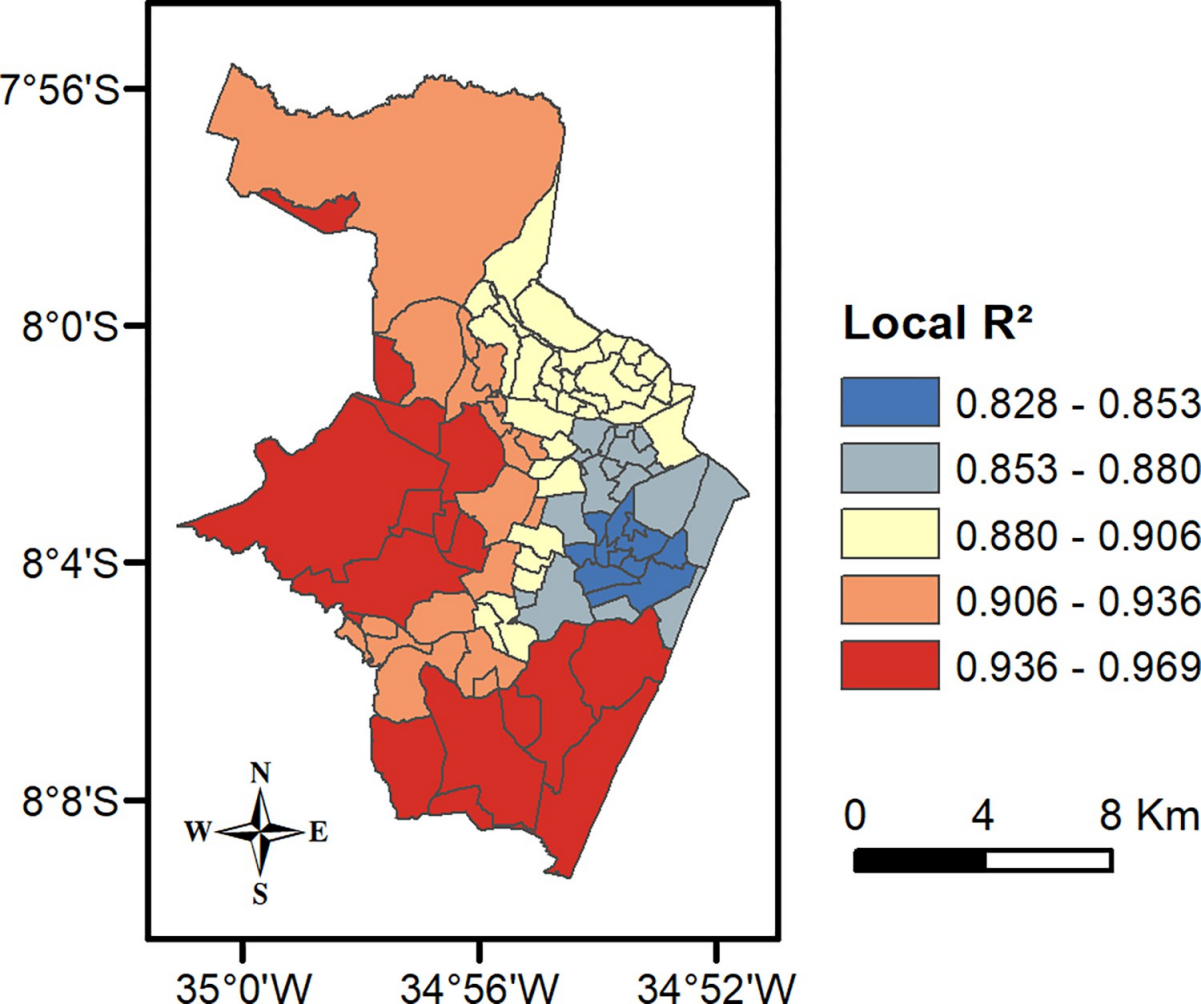
Performance of local R^2^ across the neighborhoods of Recife city. Sources: Brazilian Institute of Geography and Statistics (IBGE) 2021, and software ArcGIS 10.4.1.

GWR concerns a local prediction to elucidate spatial variations all over the region of interest [55], so the distribution of local R^2^ in each Recife neighborhood is illustrated in Fig 6. Values were found to be remarkably high since the minimum one explains 82.8% of reported cases. It was also observed that the areas with a higher local performance (the red ones) coincide with hotspots for cases (identified in Fig 4), indicating where COVID-19 infections are concentrated.

**Fig 7:**
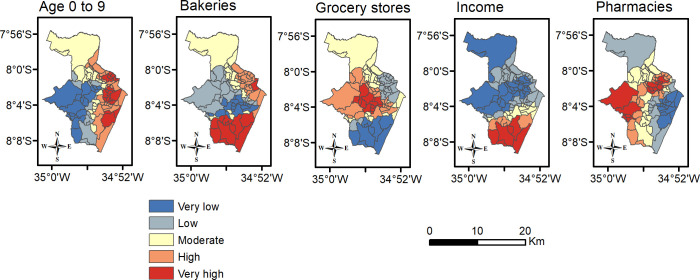
Effects of determinants on prediction of COVID-19 cases according to their coefficients in GWR regression. Sources: Brazilian Institute of Geography and Statistics (IBGE) 2021, and software ArcGIS 10.4.1.

A further exploration was made seeking to clarify how every relevant contribution of a determinant to spatial regression modelling could influence a prediction of the number of cases. Fig 7 classifies their coefficients on regression local equations into five categories by the Jenks Natural Breaks algorithm [65]. When the previously mentioned hotspots are analyzed, it is noted that the Southern ones were impacted most by the presence of bakeries and their residents’ average income. The existence of the Western cluster is best explained by the presence of local pharmacies and grocery stores. Moreover, the strongest influence on the incidence of COVID-19 in the Northern hotspot came from bakeries, pharmacies and children aged from 0 to 9.

## Discussion

Recife started applying deterrent measures in order to reduce contagion immediately after the first patients were confirmed in March 2020, thereby preventing a collapse in the provision of hospital care. After the first registered infection, cases took around a month to start a phase of sustained increase, which may have indicated an initial acceptance by the general public of these measures. Then a natural relaxation due to fatigue and ignorance about the consequences of COVID-19 was reflected in a wider local transmission of the virus. The public authorities’ initiative to tighten the quarantine had excellent results. That could be verified as the peak of contamination was reached when quarantine was still in force, after which the tendency was for the number of new cases to fall and stabilize.

Relationships concerning the explored determinants imply that some neighborhoods are generally more susceptible to the spread of COVID-19, essentially due to the direct influence of specific socioeconomic and environmental scenarios–that might be worsened when combined. COVID-19 cases are positively related to better socioeconomic conditions (household income and public services), a large population (mainly children and elderly) and the presence of some essential services (especially those connected to daily routine, such as bakeries and grocery stores). In this context, the size of the population acts as an enabler of more social contact even when social distancing measures are in force, which reiterates what was found by [16,21]. As to reported cases, hotspots were first verified in the wealthy and densely-populated neighborhood of Boa Viagem. In contrast, since then, other hotspots with worse socio-economic conditions have emerged. A similar pattern was found in the city of São Paulo, Brazil, but concerning COVID-19 deaths: what was detected was a shift of high risk from the areas with the best socio-economic conditions to those with the worst conditions [66]. This situation could have happened in Recife because, according to socio-economic indicators, a substantial proportion of the population in the Boa Viagem hotspot had the resources to travel more frequently to Brazilian metropolises, or even to other countries. Thus, these people were the first to be infected elsewhere, and, on their return to Recife, they might have contributed to spreading the disease to those around them. On the other hand, people who live in less privileged places consequently have less infrastructure in their neighborhoods, nor do their living conditions in their homes enable them to follow advice on social isolation and personal care. This includes not having enough money to buy preventive health supplies; there being a lack of constant access to piped water; and they do not have the option of working from home. All of these factors are aggravated by the social impacts of COVID-19 [67].

Bakeries, grocery stores and pharmacies seem to strongly influence the spatial spread of COVID-19 as observed in the regression analysis results. These findings were similar to a previous study in a prefecture in China, where these points-of-interest influenced the increase of the COVID-19 cluster size in the surrounding neighborhoods [68]. Going to and entering bakeries and grocery stores are part of the daily routine of regular citizens in the metropolitan region of Recife, because there is a local habit of buying fresh bread every day in the morning or late afternoon. According to a study in the city of São Paulo, Brazil, people who live in areas with a great mix of destinations within 500 m of their residences, including supermarkets, food stores and bakeries, were more inclined to walk outside home [69]. In a COVID-19 pandemic context, even during strict quarantine restrictions, residents still have to buy primary groceries and medicines, probably near to where they live. This is particularly true in low-income neighborhoods of Recife since a significant part of their population cannot afford public transportation, which restricts their routine to places they can reach on foot (or riding bicycles at most) [70]. The number of these commercial facilities is significant for predicting COVID-19, but *per se* this does not necessarily imply longer lines or crowded spaces that help to transmit SARS-CoV2. This consequence also depends on the level of demand at specific times and may be affected by the population of the neighborhood. Finally, the strong collinearity between the number of bakeries and residential population indicates that these facilities are located where the people are.

The age groups highlighted during the regression analysis reveal that places with a large number of children (between 0 and 9 years old) and/or seniors (over 60 years old) tend to present the largest number of reported cases. A similar result was found regarding the population aged above 65 at the global scale, specifically in the early weeks of the outbreak [11]. Studies affirm that most asymptomatic cases of COVID-19 are verified in children [71], so there is a high chance of their not being submitted to tests in Brazil because there is a lack of testing capacity [72]. Moreover, schools and daycare centers had been closed in Recife since mid-March 2020 [52], which stimulated those younger groups to stay at home and, therefore, it was likely that they would spread the virus to their relatives. But studies from China show that children have a lower incidence of SARS-CoV2 and are less prone than other groups to being infected by it [73]. Hence, a more in-depth exploration needs to be carried in order to determine whether this pattern also happened in Brazil, even though our results imply the opposite. On the other hand, there should be a focus on the elderly since a study specified that an increase in coronavirus infection among elderly people had a direct correlation with the risk of infections among other age groups [74]. Therefore, tightening social distancing for the elderly and other measures, such as analyzing spatial accessibility and healthcare resources [75], to reduce the risks they face could positively affect the whole of society. Furthermore, comorbidities are associated with a higher risk of severe cases of COVID-19 that demand specialized clinical care [76], so Brazilians aged over 60 need to be taken into account since they have almost 12 times higher odds of developing multiple chronic diseases than young adults [77].

Government measures to control the transmission of the virus were imposed in advance, less than a week after the first reported COVID-19 case in the state of Pernambuco. Notwithstanding, their mild severity in addition to a growing lack of society support were some of the reasons why the curve of infections did not flatten sooner. Then a 15-day milder version of the lockdown adopted in other countries was implemented in mid-May 2020, which led to a sustained tendency of incidences to fall and stabilize at a lower level. A lockdown is effective in reducing the number of new cases, particularly when it lasts for at least 10 days [78]. Our findings reaffirm what a previous study found about the positive effects of the strict quarantine in Pernambuco, which helped to increase the number of people who adopted and maintained social distancing and to reduce the reproduction rate of the virus [6].

Some factors were considered relevant for the majority of the dates examined regarding the evolution of COVID-19 cases, but were cut in regression analysis from the most recent set of determinants after the quarantine period. That was the case of lottery shops, owned home and literacy, and all of them positively affected the variability of reported cases. Lottery shops, which also act as sub-agencies of a public bank, have been used along with Caixa Econômica Federal bank agencies to make the payment of emergency aid to at least 25% of the Brazilian population since April 2020 [79]. So, the presence of these facilities could have acted as a way for promoting long queues with social distancing not being respected and physical overcrowding between socially vulnerable people [80]. For their part, literate people tend to have more information about the disease, but we cannot assume that they consider this subject as weighty and/or they may not have the resources to follow all the recommendations on isolation. This direct association of literacy diverges from other studies [15], so that our findings seem to give a distinctive characteristic of Recife’s neighborhoods. Finally, ownership of one’s own home does not point up a clear social difference among the hotspots of COVID-19 cases. This determinant was found statistically significant considering data gathered on the first and last dates examined. So it remained relevant in hotspots of cases even though the disease advanced from privileged areas to others that are historically known for under-privileged living conditions [34].

Confirmed cases remained stable or in decline for a while during the period evaluated—even after restrictive measures were relaxed -, which denoted a possible control of the pandemic. Nevertheless, at the end of 2020, Brazilian cities suffered a second wave of the COVID-19 outbreak. This was intensified as new SARS-CoV2 variants with greater transmission power started to spread worldwide [7]. Further studies should verify whether the patterns identified in the first wave of infections in 2020 have been maintained, and they should also consider the pace of vaccination campaigns.

This paper has some limitations since there is significant underreporting of COVID-19 cases and deaths in Brazil due to the limited availability of tests, and the capacity of local surveillance services. Further analysis should include more recent data to represent socio-economic characteristics, in addition to the previous health status of infected people regarding comorbidities. Finer variations in the behavior of the disease could also be captured by exploring spatial units that are even smaller than neighborhoods, such as census tracts. Another limitation refers to the influence of possible confounded variables and other extraneous variables in the model, although there is no consensus in the literature about the best strategy for dealing with them [81]. According to [82], it is equally possible that adding control variables introduces overcontrol and endogenous selection biases, thus creating alternative interpretations rather than ruling them out. Additionally, it is known that it is difficult, if not impossible, to include a comprehensive list of all factors influencing the spread of COVID-19 in a community. Thus, we believe that our findings outweigh these limitations.

## Conclusion

This study combined spatial clusters and statistical analysis to evaluate the influence of socio-economic factors and essential services on the spread of COVID-19 (in terms of reported cases and case-fatality) in the city of Recife, Brazil. Our findings reveal that an increased risk of transmissions was associated with children and the elderly, the size of the population, household income, the level of education, and the presence of some facilities that have remained open throughout the pandemic. Moreover, the spatial spread of the disease occurred by moving from well-developed to deprived neighborhoods during the initial stages of the pandemic. What was also found was for there to have been a tendency for there to have been harsh impacts (due to higher case-fatality rates) on socially vulnerable and densely populated communities, specially those with many everyday places that are prone to overcrowding (e.g. bakeries, grocery stores).

Brazil manages a public health system that is widespread in all federative units, even though the country has continental dimensions and faces complex challenges. However, currently, public agencies have been going through management difficulties. In this sense, this study can support strategic decisions to help mitigate the spread of COVID-19 not only in Brazil, but also in other developing and economically emerging countries. Furthermore, in the long term, knowledge produced during the COVID-19 pandemic in this heterogeneous context, regarding local characteristics and spatiotemporal patterns, can be used to structure policies for tackling new epidemics of viral infectious diseases.
